# New 3-Aryl-2-(2-thienyl)acrylonitriles with High Activity Against Hepatoma Cells

**DOI:** 10.3390/ijms22052243

**Published:** 2021-02-24

**Authors:** Eva Schaller, Andi Ma, Lisa Chiara Gosch, Adrian Klefenz, David Schaller, Nils Goehringer, Leonard Kaps, Detlef Schuppan, Andrea Volkamer, Rainer Schobert, Bernhard Biersack, Bianca Nitzsche, Michael Höpfner

**Affiliations:** 1 Organic Chemistry Laboratory, University of Bayreuth, Universitätsstrasse 30, 95440 Bayreuth, Germany; eva-schaller@web.de (E.S.); rainer.schobert@uni-bayreuth.de (R.S.); 2 Institute of Physiology, Charité-Universitätsmedizin Berlin, Charitéplatz 1, 10117 Berlin, Germany; andi.ma@charite.de (A.M.); lisa-chiara.gosch@charite.de (L.C.G.); nils.goehringer@charite.de (N.G.); bianca.nitzsche@charite.de (B.N.); michael.hoepfner@charite.de (M.H.); 3 In Silico Toxicology and Structural Bioinformatics, Institute of Physiology, Charité-Universitätsmedizin Berlin, Charitéplatz 1, 10117 Berlin, Germany; david.schaller@charite.de (D.S.); andrea.volkamer@charite.de (A.V.); 4 Institute of Translational Immunology, University Medical Center of the Johannes Gutenberg University, Langenbeckstrasse 1, 55131 Mainz, Germany; aklefenz@students.uni-mainz.de (A.K.); leonardkaps@googlemail.com (L.K.); detlef.schuppan@unimedizin-mainz.de (D.S.); 5 Division of Gastroenterology, Beth Israel Deaconess Medical Center, Harvard Medical School, 330 Brookline Ave, Boston, MA 02215, USA

**Keywords:** hepatoma, anticancer drugs, thiophene, tyrphostin, VEGFR inhibition, CAM assay, molecular docking

## Abstract

New 2-(thien-2-yl)-acrylonitriles with putative kinase inhibitory activity were prepared and tested for their antineoplastic efficacy in hepatoma models. Four out of the 14 derivatives were shown to inhibit hepatoma cell proliferation at (sub-)micromolar concentrations with IC_50_ values below that of the clinically relevant multikinase inhibitor sorafenib, which served as a reference. Colony formation assays as well as primary in vivo examinations of hepatoma tumors grown on the chorioallantoic membrane of fertilized chicken eggs (CAM assay) confirmed the excellent antineoplastic efficacy of the new derivatives. Their mode of action included an induction of apoptotic capsase-3 activity, while no contribution of unspecific cytotoxic effects was observed in LDH-release measurements. Kinase profiling of cancer relevant protein kinases identified the two 3-aryl-2-(thien-2-yl)acrylonitrile derivatives **1b** and **1c** as (multi-)kinase inhibitors with a preferential activity against the VEGFR-2 tyrosine kinase. Additional bioinformatic analysis of the VEGFR-2 binding modes by docking and molecular dynamics calculations supported the experimental findings and indicated that the hydroxy group of **1c** might be crucial for its distinct inhibitory potency against VEGFR-2. Forthcoming studies will further unveil the underlying mode of action of the promising new derivatives as well as their suitability as an urgently needed novel approach in HCC treatment.

## 1. Introduction

Liver cancer (hepatoma) can be subdivided into hepatocellular carcinoma (HCC, the prevalent form of hepatoma) and hepatoblastoma (which mainly affects young children) [1,2]. Treatment is only curative for earlier stages of HCC, and viable options are liver resection, local radio/chemoablation, and transplantation [2,3]. Chemotherapy has limited efficacy in cases of underlying liver cirrhosis, and due to intrinsic chemoresistance of most HCCs [4]. Tyrosine kinase receptors are involved in tumorigenesis; therefore, kinase inhibitors show some benefit and, to date, the multikinase inhibitors sorafenib, lenvatinib and regorafenib have been approved for the treatment of HCC [5,6,7]. However, the overall survival of the majority of HCC patients who are not eligible for curative approaches is unfavorable [8]. Thus, new efficient drugs against HCC that may also be used in combination therapies are urgently needed.

Heterocyclic compounds play a prominent role in the design of new drug candidates [9]. Among the sulfur-containing heterocycles, the heteroaromatic thiophene (C_4_H_4_S, from Greek “theion”, “sulfur” and “phaino”, “shining”) has emerged as a promising structural scaffold for the design of new pharmacologically active compounds [10]. Disclosed examples of antitumor active thiophenes comprise chalcones and thienopyrimidines [11,12,13,14]. The Knoevenagel reaction is a straightforward method to form C=C bonds and aryl-substituted acrylonitriles can be easily prepared by Knoevenagel reaction from aryl acetonitriles and aryl aldehydes [15,16]. Compounds of this type (so-called tyrphostins, short for: tyrosine kinase phosphorylation inhibitors) have shown promising antitumor effects by inhibiting the tyrosine kinase activity of growth factor receptors such as the epidermal growth factor receptor (EGFR) [17]. (Arene)Ru(II) complexes of such tyrphostins showed increased selectivity and anti-proliferative activity against tumor cells including HCC cells [18]. Several antitumor active derivatives were disclosed bearing aminophenyl, halophenyl or heterocyclic residues [19,20,21,22,23,24,25]. Promising thienyl derivatives were prepared in this way [26]. Thienyl-substituted compounds were identified as potent drug efflux inhibitors or as antitumor active compounds in their own right [27,28].

In this work, we present new thienyl-substituted acrylonitriles as highly active compounds against hepatocellular carcinoma cells. The effects of aryl substituents such as hydroxy and alkoxy groups, dialkyl amines, and halogens, on antiproliferative activity, apoptosis induction, and protein kinase inhibition were studied by biological and bioinformatic evaluations giving a first insight into their antineoplastic efficacy and mode of action.

## 2. Results

### 2.1. Chemistry

**Scheme 1:**
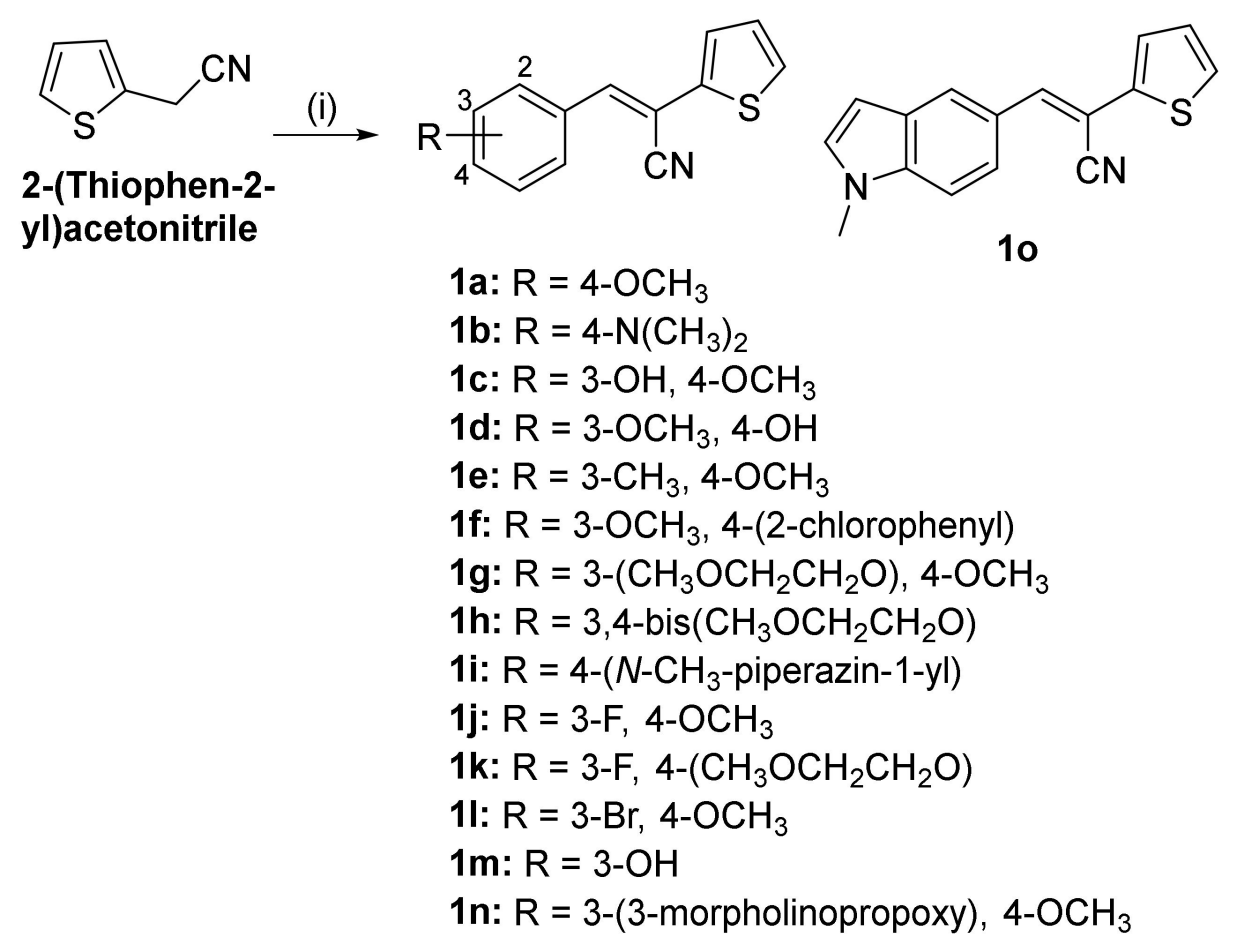
Reagents and conditions: (**i**) aryl aldehyde, cat. piperidine, EtOH, reflux, 24–48 h, 30–46%.

**Scheme 2:**
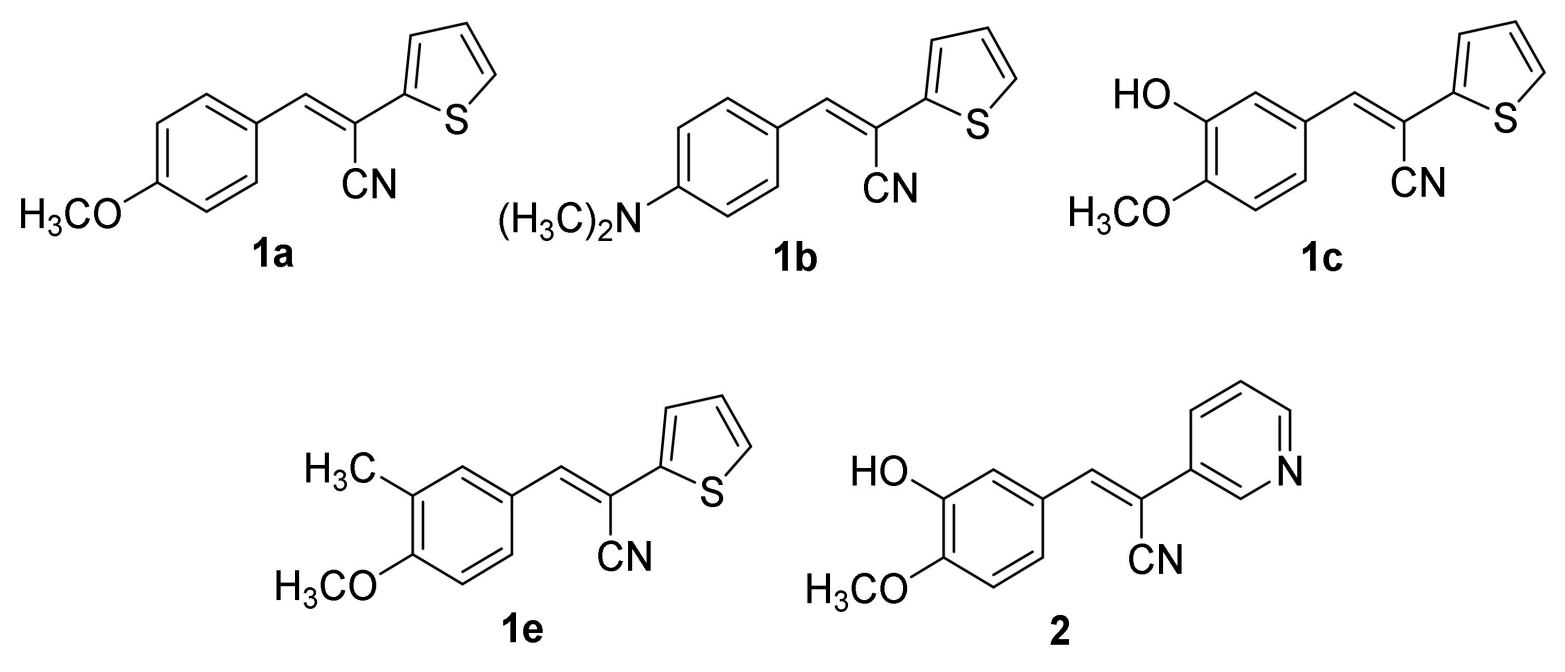
Structures of compounds **1a**, **1b**, **1c**, and **1e**, which were selected for mechanistic studies, and of the known pyridyl analog **2**.

Compounds 2-(Thien-2-yl)acrylonitriles **1a**–**o** were prepared by a Knoevenagel reaction of 2-(thiophen-2-yl)acetonitrile with the corresponding aryl aldehyde and catalytic amounts of piperidine in hot ethanol in moderate yields of 30–46% (Scheme 1). Compounds **1a** and **1b** were already published before, while the compounds **1c**–**o** were new [26,29]. Compounds **1b**, **1c**, **1e**, **1f**, **1i**, **1j**, **1k**, **1l**, **1m**, and **1o** were obtained as yellow solids, and compounds **1a**, **1d**, **1g**, **1h**, and **1n** as yellow or brown oils or gums. It is difficult to determine *E* and/or *Z* configuration of the test compounds by NMR spectroscopy. They are probably not a mixture of *E* and *Z*. Quiroga et al. have identified the *E* configuration of their derivatives of the type of compounds **1** by X-ray crystal structure experiments for one selected compound [28]. Hence, compounds **1a**–**o**, which are presented in this manuscript, are presumably *E*-derivatives, too (Scheme 1). Analogously, the known pyridine derivative **2**, which was used as a reference compound in this study, is probably the *Z*-isomer (Scheme 2) [18,29].

### 2.2. Biological Evaluation

The compounds **1a**–**o** were studied for their anti-proliferative activities against human HepG2 hepatoblastoma cells, human Huh-7 HCC cells, and non-transformed hepatocytes (AML-12) (Table 1). Out of the tested 14 derivatives, four (**1a**, **1b**, **1c**, and **1e**) showed pronounced growth inhibitory effects both in p53-wildtype (HepG2) and p53-mutated (Huh-7) hepatoma cells with IC_50_ values in the (sub-)micromolar range, thereby being below that of the clinically relevant inhibitor sorafenib, which was used as a reference (Table 1). The new derivative **1c** exhibited the highest activities of this whole series of thiophenes (IC_50_ = 0.55 µM for HepG2, 0.32 µM for Huh-7) and **1c** was also more active than the known derivatives **1a** and **1b**. It is interesting to note that **1d**, the isomer of **1c**, displayed no activity at concentrations up to 20 µM. The new 3-methyl analog **1e** was roughly as active as **1a**. In addition, the 3-fluoro derivative **1j** (IC_50_ = 4.90 µM for HepG2, 5.70 µM for Huh-7) and the *N*-methylpiperazine derivative **1i** (IC_50_ = 9.30 µM for HepG2, 11.79 µM for Huh-7) displayed moderate activities against these two hepatoma cell lines. The bromo derivative **1l** was distinctly less active than its fluoro congener **1j**. Although the replacement of the 3-hydroxy group by methyl or fluorine still led to active compounds, the etherification with 2-methoxyethyl or 3-morpholinopropyl residues formed virtually inactive compounds **1g**, **1h**, and **1n** as far as anti-proliferative activity is concerned. Compound **1f** with a chlorophenyl substituent was likewise inactive. Compound **2** (see Scheme 2), the known 3-pyridyl analog of **1c**, was much less active than thiophene **1c** [18,29]. The most active compounds **1a**, **1b**, **1c**, and **1e** showed a reduced anti-proliferative activity against non-malignant AML-12 hepatocytes, with IC_50_ values that were up to 10-fold higher than those against hepatoma cells, indicating a certain tumor cell specificity for the hepatoma cells. Hence, these compounds were selected for further studies concerning modes of anticancer action (Scheme 2).

**Figure 1:**
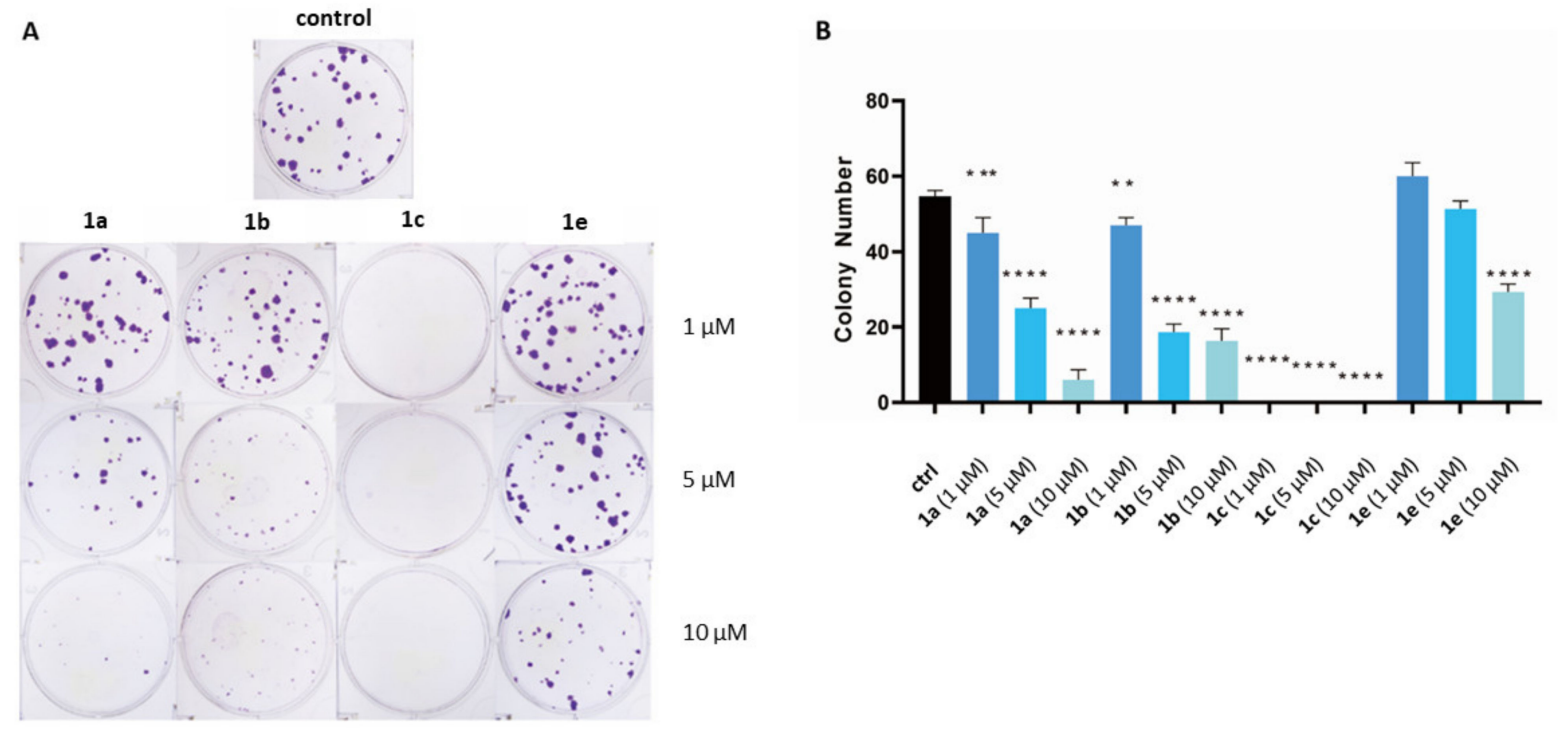
Left figure: Colony formation assays revealed a dose-dependent inhibition of HepG2 colony formation after 14 days of incubation with **1a**, **1b**, **1c** and **1e**. Right figure: mean values ± SD of colony numbers of *n* = 3 experiments. Statistical significance was tested by one-way ANOVA, ** *p* < 0.01, *** *p* < 0.001, **** *p* < 0.0001.

Long term effects of **1a**, **1b**, **1c** and **1e** on hepatoma growth and spreading were investigated by colony formation assays, in which a colony was defined as a cell aggregate of 50 or more cells [30]. Colony formation of HepG2 cells was assessed over a period of 14 days. As shown in Figure 1, all four derivatives significantly inhibited HepG2 colony formation in a dose-dependent manner, with **1c** being the most effective compound with almost 100% inhibition of colony formation after two weeks. Moreover, the size of the remaining colonies dramatically decreased, further indicating the high antiproliferative efficacy of the derivatives.

**Figure 2:**
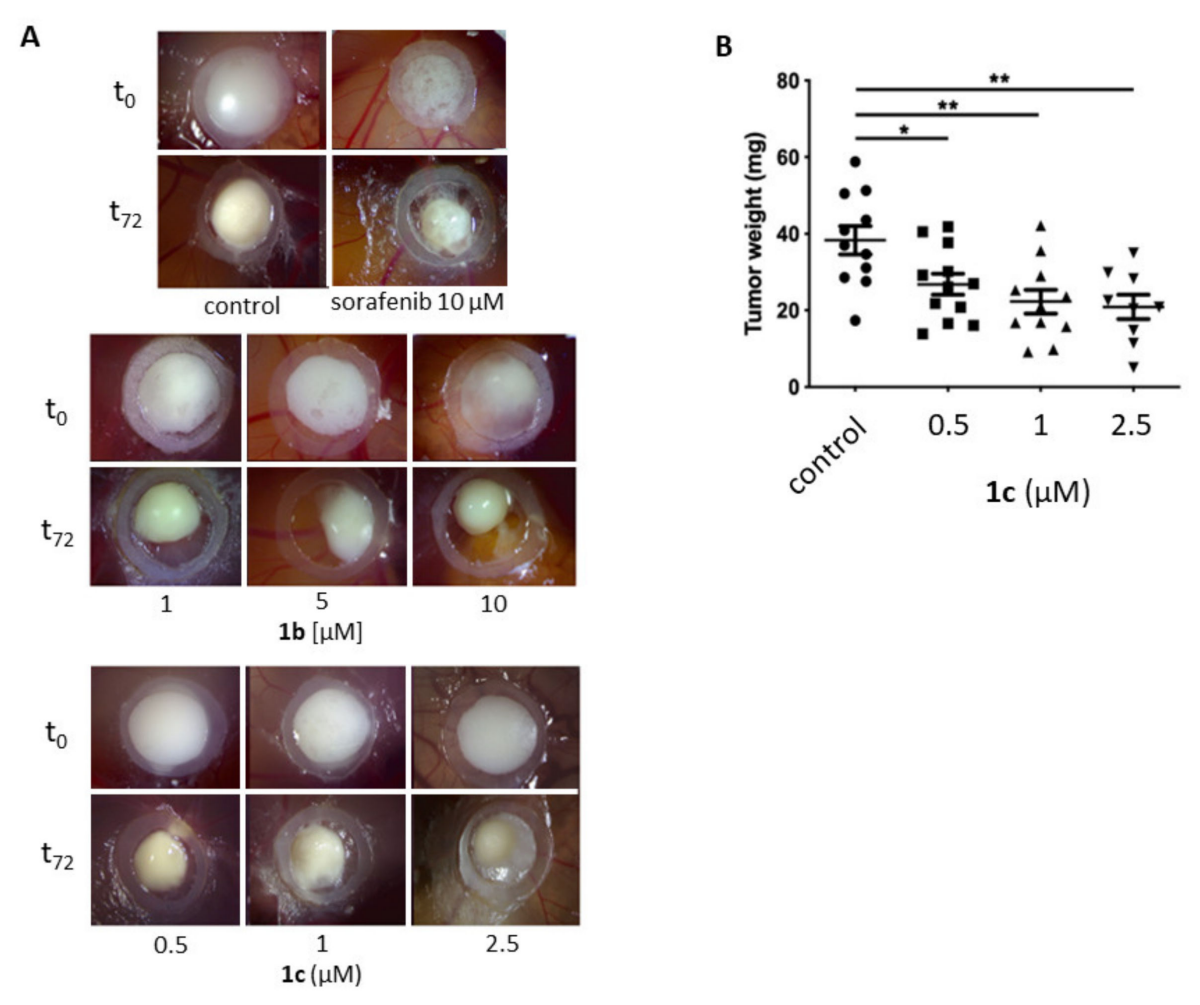
Suppression of hepatoma tumor growth in vivo. (**A**) Representative images of hepatoma tumor bearing CAMs at the beginning (t_0_) and after 72 h (t_72_) of treatment with **1b** (middle panel) and **1c** (lower panel). Growth of PBS- and sorafenib-treated tumors served as control and sorafenib-reference, respectively (upper panel), *n* = 2 experiments for **1b** and *n* = 3 experiments for **1c**. (**B**) Statistical analysis of the effects of **1c** revealed a dose-dependent and significant decrease in tumor weight after 3 days of treatment. Box plot diagram indicates mean tumor weight ± SD for each condition. Significance was determined by one-way ANOVA, * *p* < 0.05, ** *p* < 0.01.

For a first impression of the antineoplastic efficacy of the new derivatives in a systemic scenario, we additionally checked the effects of **1b** and **1c** in xenograft experiments of HepG2-tumors grown on membranes of fertilized chicken eggs (CAM assay). HepG2-matrigel tumor plugs with 3 × 10^6^ HepG2 cells inoculated onto the CAM of 10-day-old, fertilized chicken eggs were allowed to attach and connect to the CAM vascular network for 24 h. Subsequent topical treatment with rising concentrations of **1b** and **1c** or 10 µM sorafenib led to pronounced tumor reductions, as compared to PBS-treated controls (Figure 2). The effects of **1b** and **1c** were dose-dependent, and even exceeded that of sorafenib-treated tumors. Extraction and weighing the tumors after treatment revealed a significant mass reduction in **1c**-treated tumors. The treatment was well tolerated; no increased mortality or delayed development of the chicken embryos was observed in treated vs. untreated eggs.

**Figure 3:**
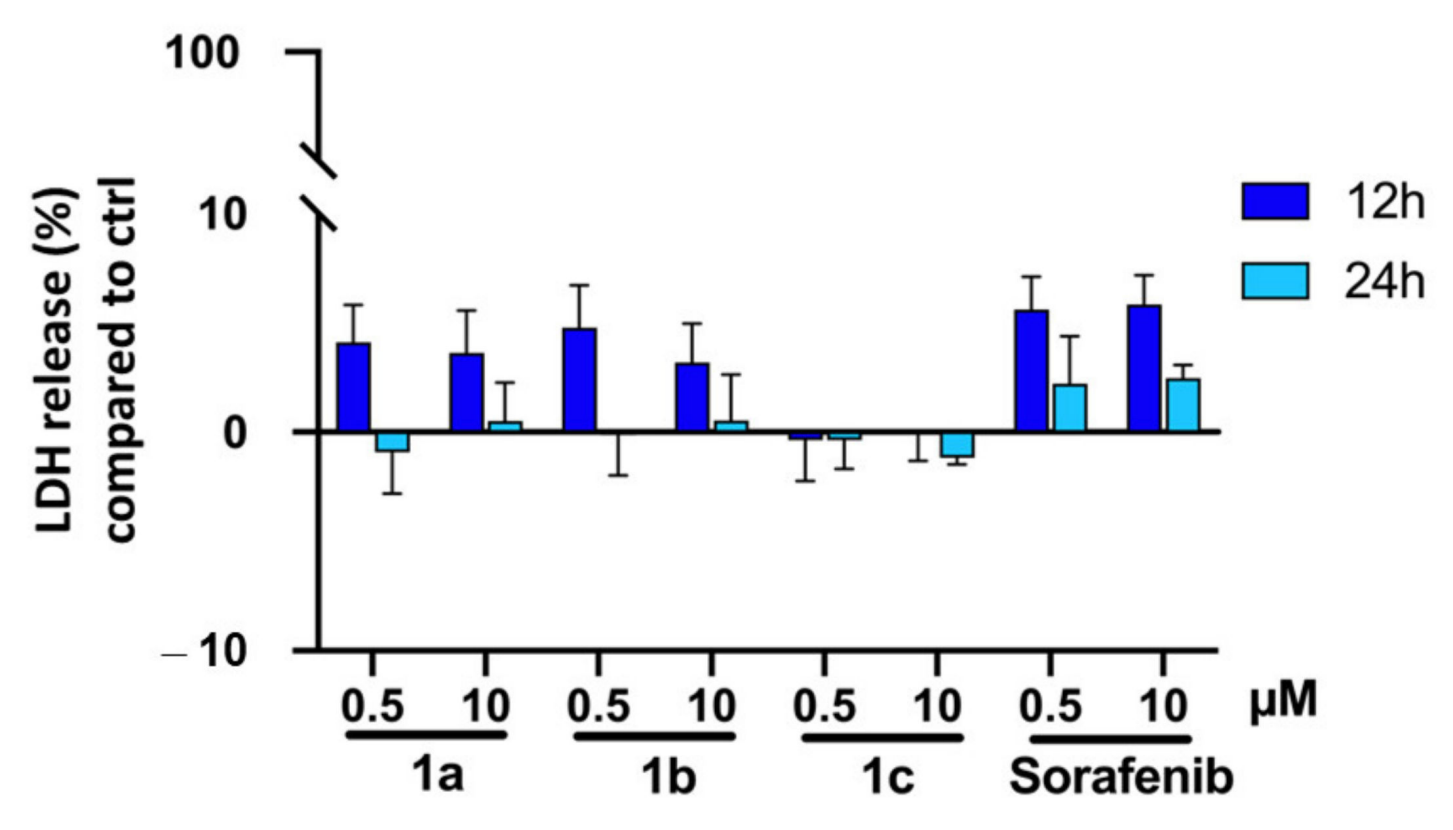
Cytotoxic effects **1a**, **1b**, **1c** and sorafenib. Release of lactate dehydrogenase (LDH) after incubation of HepG2 cells with 0.5 and 10 µM of **1a**, **1b**, **1c** and sorafenib was not significantly altered when compared to untreated controls (set to 0%), indicating that unspecific toxicity did not contribute to the observed effects. Means ± SD of *n* = 3 independent experiments.

To confirm that the antiproliferative effects of the new derivatives were not based on the induction of unspecific cytotoxicity, **1a**, **1b**, **1c** and sorafenib were tested in lactate dehydrogenase (LDH) release assays, in which treatment-induced increases in LDH-release into the supernatant of cell cultures indicate unspecific and immediate cytotoxic damage of cell membranes and organelles. However, as depicted in Figure 3 neither low nor high concentrations of **1a**, **1b**, **1c** and sorafenib substantially increased the LDH release of HepG2 cells after 12 or 24 h and, thus, do not exert considerable unspecific cytotoxicity.

**Figure 4:**
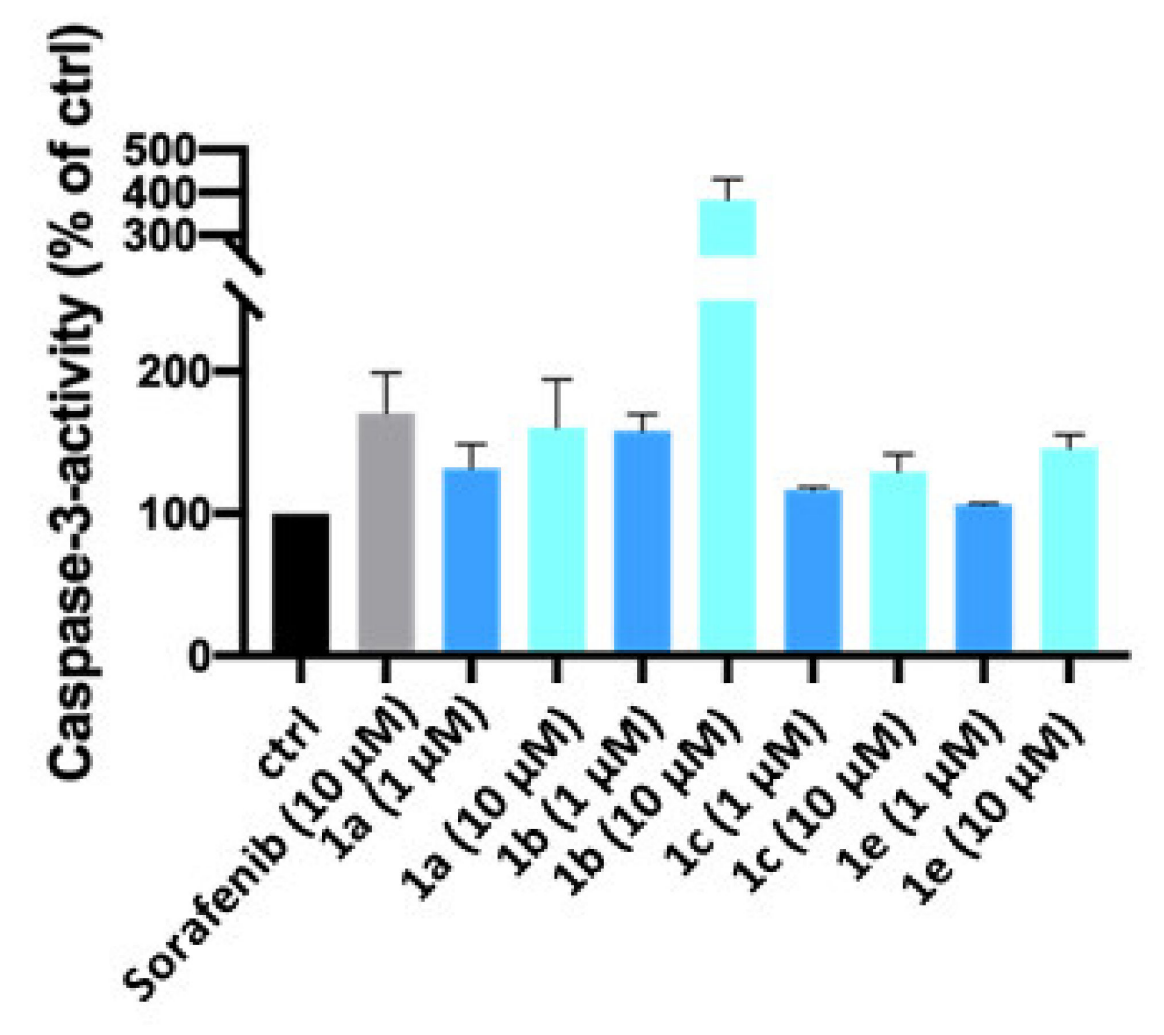
Induction of apoptotic caspase-3 activity. Treatment with **1a**, **1b**, **1c**, **1e** and sorafenib for 24 h dose-dependently induced caspase-3 activity of HepG2 cells. Data of *n* = 3 experiments are shown as means ± SD of percentage increase as compared to untreated controls.

The ability to induce apoptosis was investigated for the most active compounds **1a**, **1b**, **1c** and **1e** (Figure 4). Apoptosis specific caspase-3 activity of HepG2 cells was shown to be induced after 24 h of treatment. The induction caused by the new derivatives was in the same range as that of sorafenib, which was used as a reference. Interestingly, at a concentration of 10 µM, compound **1b** induced a comparably stronger increase in caspase-3 activity than the other compounds. However, further evaluations on the apoptotic effects of the new derivatives are required and will take the time-dependency and involvement of mitochondria-driven apoptosis into account.

### 2.3. Enzymatic Kinase Assays

**Figure 5:**
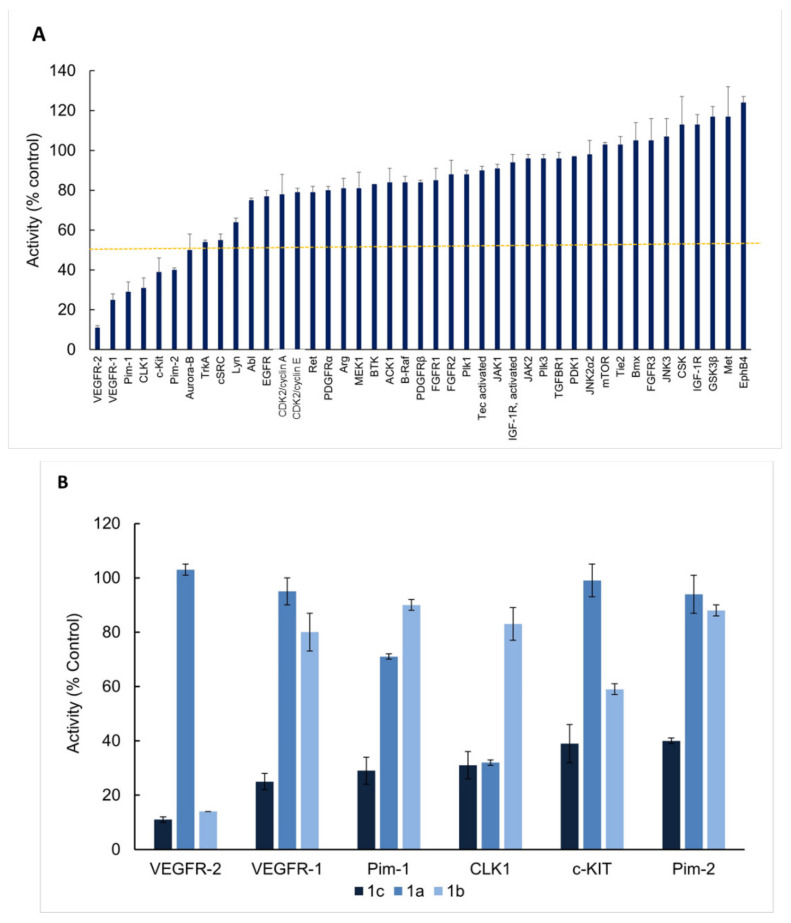
(**A**) Enzymatic assay screening results for compound **1c** on a panel of 43 kinases. (**B**) Screening results for compounds **1a**, **1b** and **1c** against a subpanel of six protein kinases. Kinase profiling was performed in duplicate with 10 µM of either compound employing Eurofins KinaseProfiler TM service. Data are given as mean values ± SD of kinase activity in %, as compared to non-treated kinases (100%).

Synthesized as putative tyrosine kinase inhibitory compounds, the best working derivatives **1a**, **1b** and **1c** were tested for their inhibitory activity against a panel of protein kinases consisting of 43 kinase targets that were preselected by tumor relevance and in silico target prediction (based on the lead compound **1c**). Compound **1c** inhibited six out of the 43 putative kinase targets by more than 50% in an enzymatic kinase assay. Compound **1c** displayed considerable selectivity for VEGFR-2 (KDR), which was inhibited by 89% (Figure 5A). The IC_50_ value of **1c** against VEGFR-2 amounted to 3.32 µM. Compound **1c** also strongly inhibited VEGFR-1 by 75% as well as c-KIT (61% inhibition), while it showed no relevant inhibition for other growth factor receptor tyrosine kinases such as EGFR, IGF-1R, PDGFR or FGFR. Other kinases that were inhibited by more than 50% by **1c** included CLK1 (69% inhibition), Pim-1 (71% inhibition) and Pim-2 (60% inhibition) (Figure 5B). Thus, **1c** appeared to be a multikinase-inhibiting compound with pronounced VEGFR-2 selectivity.

Based on these findings, compounds **1a** and **1b** were also screened against the subpanel of the six most affected kinases detected in the panel screening with compound **1c**. In contrast to **1c**, **1a** and **1b** showed a rather selective inhibition of mainly one of the six kinases of the subpanel (Figure 5B). While compound **1a** inhibited CLK1 by 68%, compound **1b** displayed a high selectivity for VEGFR-2 which was inhibited by 86%, and thus was almost as potent as **1c**.

### 2.4. Molecular Modeling

To rationalize the structure-activity relationship of **1a**, **1b** and **1c** with the kinase VEGFR-2, the compounds were investigated for a potential common binding mode by performing molecular dockings. Inhibitors of protein kinases are known to prefer binding to distinct kinase conformations, which could heavily affect bioinformatic docking studies [31]. An important motif used to determine kinase conformations harbors the amino acid sequence Asp-Phe-Gly (DFG) and is found in the beginning of the activation loop. Kinase conformations with the aspartic acid of the DFG motif pointing inside the ATP binding pocket are referred to as DFG-in and are considered to represent the active ATP binding competent kinase conformation. All available human VEGFR-2 structures in the DFG-in conformation lack large portions of the important activation loop and many VEGFR-2 inhibitors bind to a DFG-out (inactive) conformation [32]; therefore, our efforts focused on human VEGFR-2 structures in the DFG-out conformation. The DFG-out X-ray structure with the best resolution (3VHE) [33] was selected for conducting molecular docking and molecular dynamics simulations.

**Figure 6:**
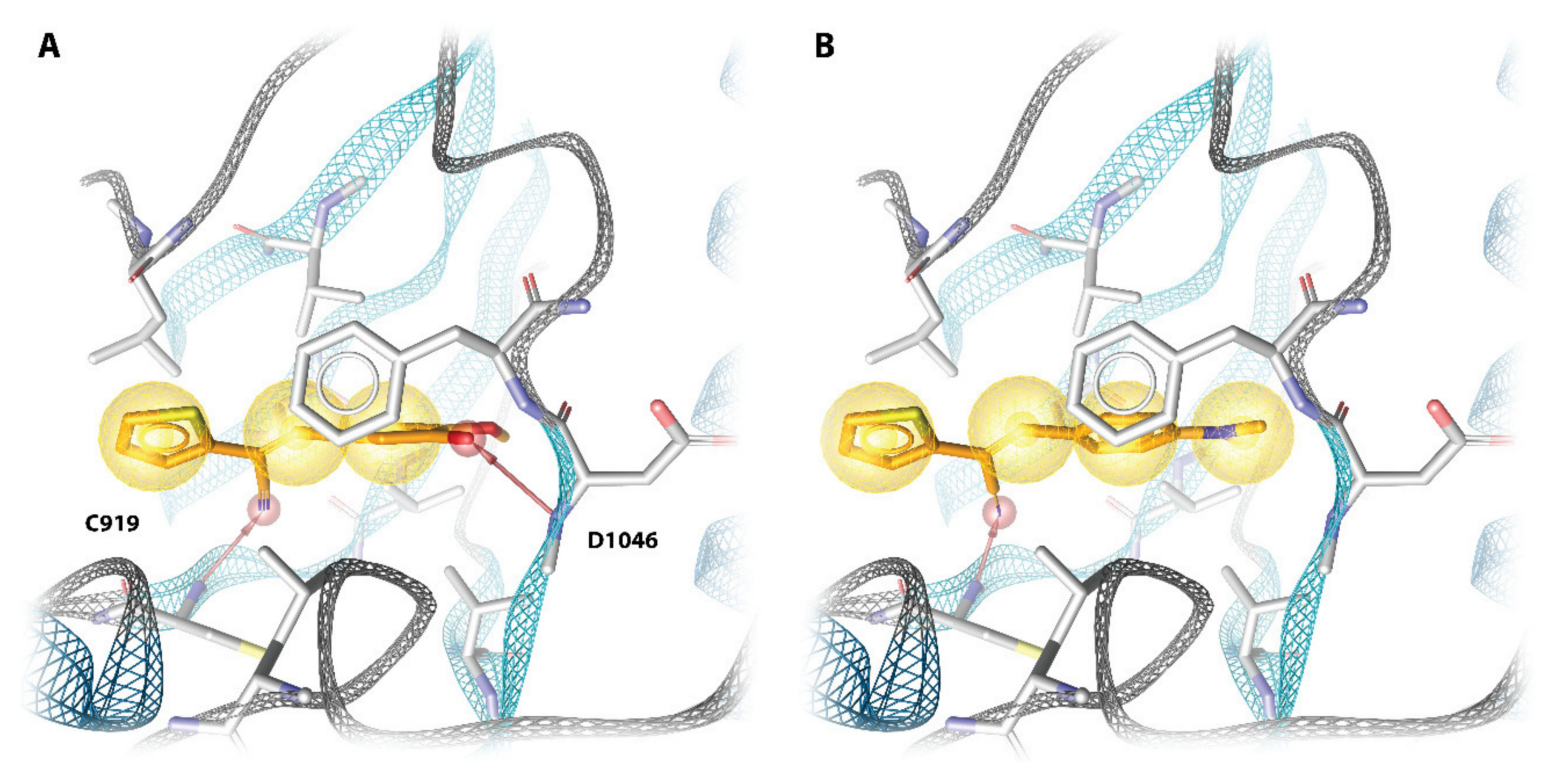
Identified binding mode of (**A**) compound **1c** and (**B**) compound **1b** against human VEGFR-2 structure 3VHE. The protein cartoon representation is colored light blue for beta-sheets, dark blue for helices, and grey for loop structures. Ligands are depicted as sticks with carbon atoms colored in orange. Highlighted protein residues are involved in interactions with both compounds and are depicted as sticks with white carbon atoms. Red arrows, hydrogen bond acceptors; yellow spheres, hydrophobic contacts.

Molecular docking was performed using the SeeSAR software version 10.2. Selected poses were subjected to energy minimization with the MMFF94s force field [34] and visual inspection in LigandScout 4.4 [35]. The most consistent binding mode among the three docked compounds involved a hydrogen bond with the backbone nitrogen of cysteine C919, as well as several hydrophobic contacts as identified with LigandScout 4.4 [35] (Figure 6). The hydrogen bond between the nitrile group and C919 of the hinge region of the kinase is an interaction commonly observed for protein kinases. The most active compound in the enzymatic assay **1c** exclusively holds a hydroxyl group in meta position, making a favorable hydrogen bonding interaction with the backbone nitrogen of aspartic acid D1046 from the DFG motif, which could explain its superior activity compared to **1a** and **1b** (Figure 6A). Surprisingly, while compounds **1a** and **1b** have no hydroxyl group in meta position only compound **1a** lacks any activity at a testing concentration of 10 µM. However, compound **1b** bears a dimethylamino group in para position allowing additional hydrophobic contacts, which could explain its remaining activity against VEGFR-2 (Figure 6B).

**Figure 7:**
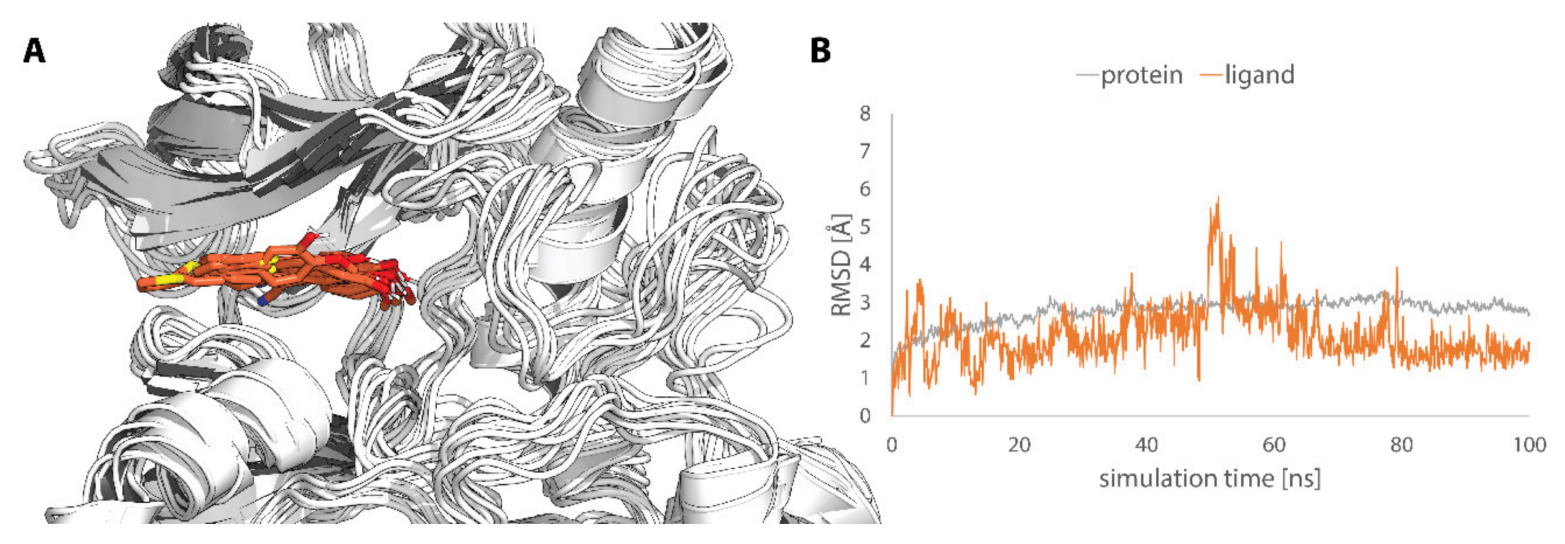
Results of a 100 ns MD simulation of VEGFR-2 in complex with compound **1c** in the proposed binding mode. (**A**) Snapshots of the MD simulation taken every 10 ns. Compound **1c** is depicted with sticks and carbon atoms colored in orange. (**B**) Root-mean-square deviation (RMSD) as a measure of the average distance between heavy atoms of ligand and protein, respectively.

To further investigate the identified binding mode, we performed an unrestrained 100 ns molecular daynamics (MD)simulation of VEGFR-2 in complex with the lead compound **1c** using Desmond 6.1 (Schrödinger Suite 2020-1) (Figure 7A). Although the ligand alters its binding mode after 50 ns, it quickly returns to its initial binding pose, which further emphasizes the validity of the proposed binding mode (Figure 7B).

## 3. Discussion

The new 3-[(3-hydroxy-4-methoxy)phenyl]-2-(thien-2-yl)acrylonitrile **1c** was identified as a potent inhibitor of hepatoma cell proliferation. This compound was more active than known kinase inhibitors such as gefitinib, sorafenib and the known pyridyl analog **2** against HCC cells [36]. The anti-hepatoma activity of **1c** in comparison with that of **2** is remarkable because we showed before that **2** is active against cancer cells of other tumor cell lines such as HL-60 leukemia and multidrug resistant breast and cervix carcinoma cells [18]. It is conceivable that the activity of **1a** is in parts based on metabolic oxidation/hydroxylation to **1c**, because this was already observed for similar pyridine analogs [29]. Hence, **1a** could be a prodrug of **1c**. Compound **1c** killed hepatoma cells by the induction of apoptosis and it exhibited no considerable unspecific cytotoxicity. The time point for the determination of caspase-3 activity after 24 h was deduced from the literature [37]. Comparing the new compounds with sorafenib, caspase-3 activity was checked based on kinetics that have already been reported for sorafenib in Hep-G2 cells, showing that sorafenib most potently induced caspase-3 activity after 24 h, while it markedly dropped after 48 h.

The one-step preparation of **1c** from commercially available inexpensive starting materials contributes to its attractiveness. In addition, prodrug derivatization strategies to protect the hydroxy group of **1c** from metabolic reactions appear promising.

Compound **1c** exhibited multikinase inhibitory potency with considerable selectivity for VEGFR-2. VEGFR inhibitors as anti-angiogenic agents are widely used as a target-specific treatment option for the management of hepatoma and of other cancer diseases such as renal cell carcinoma (RCC) and gastrointestinal stromal tumor (GIST) [38]. Thienyl-based VEGFR-2 inhibitors have been published, such as chalcone derivatives with thienyl moieties, which showed considerable VEGFR-2 inhibitory activities as well as activities against HUVEC cells as an angiogenesis cell model [14]. Several works about new VEGFR-2 inhibitors were disclosed, which indicate the clinical potential and importance of this research field [39,40,41]. Compound **1c** also inhibited the receptor tyrosine kinase c-Kit, which is a cell growth promoting factor and a relapse risk enhancer in hepatoma [42].

Our molecular modeling efforts involving molecular docking and molecular dynamics studies identified a common binding mode for compounds **1b** and **1c** at VEGFR-2. This binding mode was stable over a 100 ns unrestrained MD simulation for the most active compound **1c**. Interestingly, compound **1a** did not show significant activity against VEGFR-2 in the enzymatic assay, although it only lacked the hydroxyl group in meta position when compared to compound **1c**. In contrast, compound **1b**, which also lacks this hydroxyl group, showed considerable activity in the enzymatic kinase testing of VEGFR-2. However, compound **1b** features a dimethylamino group instead of the methoxy residue of compound **1c**. Hence, we speculate that exchanging the methoxy group of **1c** with the dimethylamino group of **1b** might lead to an even more potent inhibition of VEGFR-2 kinase activity.

In addition to VEGFR-1 and -2, compound **1c** also potently inhibited Pim-1. Pim-1 is a serine–threonine kinase and its inhibition suppressed cytokine formation as well as cell proliferation and survival [43]. Sorafenib, for instance, shows no Pim-1 inhibitory activity [44]. Thus, thiophene **1c** appears to be a promising drug candidate worth further in-depth investigation concerning its activity and exact mode of action, potentially complementing the activity of other VEGFR blockers such as sorafenib, against which resistance mutations can quickly arise.

## 4. Materials and Methods

### 4.1. General Procedures

Melting points were measured with a Gallenkamp apparatus (uncorrected, Electrothermal, Stone, UK). IR spectra were obtained from a PerkinElmer Spectrum One FT-IR spectrophotometer with ATR (attenuated total reflection) sampling unit. NMR spectra were obtained from a BRUKER Avance 300 spectrometer and chemical shifts (δ) are given in parts per million downfield from tetramethylsilane as the internal standard. The ^1^H NMR signals are described as s (singlet), br s (broad singlet), d (doublet), dd (doublet of doublets), t (triplet) and m (multiplet). Coupling constants J (in Hz) are provided. Mass spectra were measured on a Varian MAT 311A (EI). For HRMS (high-resolution mass spectrometry) analyses, an UPLC/Orbitrap system in ESI mode was applied. Microanalyses were obtained from an Elementar Unicube and test compounds were >95% pure by elemental analyses.

### 4.2. Materials

Chemicals and reagents were purchased from Sigma-Aldrich (Darmstadt, Germany): 2′-Chloro-2-methoxy-[1,1′-biphenyl]-4-carboxaldehyde, 4-methoxy-3-(2-methoxyethoxy)-benzaldehyde, 4-(4-methylpiperazin-1-yl)-benzaldehyde, 3,4-bis(2-methoxyethoxy)-benzaldehyde, 3-fluoro-4-(2-methoxyethoxy)-benzaldehyde, and 4-methoxy-3-(3-morpholinpropoxy)-benzaldehyde were synthesized as published [45,46,47,48,49]. The known compounds **1a**, **1b**, and **2** were prepared according to a literature procedure, and the analytical data of **1a**, **1b**, and **2** used in this study were in line with the published data [18,26,28,29].

### 4.3. Synthesis of Compounds **1c**–**o**

Compound 2-[1-Cyano-2-(3-hydroxy-4-methoxyphenyl)-(E)-ethenyl)]-thiophene (**1c**) was synthesized by a typical procedure: 3-Hydroxy-4-methoxybenzaldehyde (502 mg, 3.3 mmol) was dissolved in ethanol (p.a., 10 mL) and 2-thiopheneacetonitrile (350 µL, 3.3 mmol) was added. A catalytic amount of piperidine (eight drops via a Pasteur pipette) was given, and the reaction mixture was vigorously stirred under reflux for 24–48 h. After cooling to room temperature, the solvent was evaporated and the residue was taken up in ethyl acetate, washed with water, and dried over Na_2_SO_4_. The solvent was evaporated, and the residue was recrystallized from EtOH/H_2_O after hot filtration or purified by column chromatography (silica gel 60). Yield: 333 mg (1.29 mmol, 39%); yellow solid of mp 113 °C; *R*_f_ = 0.25 (ethyl acetate/*n*-hexane, 1:3); *ν*_max_ (ATR)/cm^−1^: 3460, 3106, 3011, 2932, 2849, 2217, 1616, 1597, 1573, 1508, 1474, 1462, 1438, 1430, 1376, 1351, 1337, 1274, 1240, 1212, 1180, 1130, 1053, 1001, 975, 920, 897, 860, 845, 826, 793, 779, 755, 705; ^1^H NMR (300 MHz, CDCl_3_): δ 3.90 (3 H, s, OCH_3_), 5.79 (1 H, s, OH), 6.87 (1 H, d, J = 8.5 Hz, Ph-5H), 7.0–7.1 (1 H, m, thienyl-4H), 7.2–7.5 (5 H, m, Ph/thienyl-H, CH); ^13^C NMR (75.5 MHz, CDCl_3_): δ 55.9 (OCH_3_), 103.6 (*C*-CN), 110.6, 114.8, 117.1 (CN), 122.4, 125.6, 126.4, 126.8, 127.9, 139.4, 139.5, 145.7 (COH), 148.5 (*C*-OCH_3_); *m*/*z* (%) 257 (82) [M^+^], 242 (7), 224 (17), 214 (18), 196 (100), 186 (11), 115 (12); HRMS: 258.05851 (calcd. 258.05833) [M^+^ + H].

Compound 2-[1-Cyano-2-(4-hydroxy-3-methoxyphenyl)-(*E*)-ethenyl)]-thiophene (**1d**): analogously to the synthesis of **1c**, compound **1d** was obtained from 4-hydroxy-3-methoxybenzaldehyde (502 mg, 3.3 mmol), 2-thiopheneacetonitrile (350 µL, 3.3 mmol) and a catalytic amount of piperidine (eight drops via a Pasteur pipette) in ethanol (p.a., 10 mL). Yield: 280 mg (1.09 mmol, 33%); yellow oil; *R*_f_ = 0.50 (ethyl acetate/*n*-hexane, 1:2); *ν*_max_ (ATR)/cm^−1^: 3361, 3101, 3013, 2939, 2842, 2213, 1579, 1508, 1463, 1428, 1380, 1342, 1279, 1234, 1203, 1183, 1160, 1126, 1054, 1029, 975, 893, 847, 808, 695; ^1^H NMR (300 MHz, CDCl_3_): δ 3.96 (3 H, s, OCH_3_), 5.97 (1 H, s, OH), 6.95 (1 H, d, J = 8.3 Hz, Ph-5H), 7.0–7.1 (1 H, m, thienyl-4H), 7.2–7.4 (4 H, m, Ph/thienyl-H), 7.67 (1 H, s, CH); ^13^C NMR (75.5 MHz, CDCl_3_): δ 56.1 (OCH_3_), 103.0 (*C*-CN), 109.9, 114.7, 117.5 (CN), 125.2, 125.5, 126.0, 126.4, 128.0, 139.6, 139.9, 146.7 (COH), 148.2 (*C*-OCH_3_); *m*/*z* (%) 257 (100) [M^+^], 242 (3), 224 (7), 214 (11), 196 (46), 186 (7), 115 (5).

Compound 2-[1-Cyano-2-(3-methyl-4-methoxyphenyl)-(*E*)-ethenyl)]-thiophene (**1e**): analogously to the synthesis of **1c**, compound **1e** was obtained from 3-methyl-4-methoxybenzaldehyde (496 mg, 3.3 mmol), 2-thiopheneacetonitrile (350 µL, 3.3 mmol) and a catalytic amount of piperidine (eight drops via a Pasteur pipette) in ethanol (p.a., 10 mL). Yield: 237 mg (0.98 mmol, 30%); yellow solid of mp 119–120 °C; *R*_f_ = 0.68 (ethyl acetate/*n*-hexane, 1:2); *ν*_max_(ATR)/cm^−1^ 3105, 3091, 3070, 3018, 2966, 2930, 2900, 2834, 2215, 1601, 1572, 1506, 1520, 1506, 1464, 1449, 1431, 1406, 1382, 1346, 1319, 1299, 1259, 1229, 1190, 1132, 1093, 1055, 1027, 1000, 962, 901, 865, 843, 802, 777, 763, 747, 723, 716, 701, 632; ^1^H NMR (300 MHz, CDCl_3_) δ 2.24 (3 H, s, CH_3_), 3.86 (3 H, s, OCH_3_), 6.86 (1 H, d, J = 8.6 Hz, Ph-5H), 7.0–7.1 (1 H, m, thienyl-4H), 7.2–7.3 (3 H, m, Ph/thienyl-H), 7.63 (1 H, s, CH), 7.74 (1 H, d, J = 8.6 Hz, Ph-6H); ^13^C NMR (75.5 MHz, CDCl_3_) δ 16.2 (CH_3_), 55.4 (OCH_3_), 102.8 (*C*-CN), 110.0, 117.4 (CN), 125.0, 125.3, 125.6, 125.9, 126.2, 127.2, 127.3, 127.9, 128.6, 131.7, 139.8, 159.7 (*C*-OCH_3_); *m*/*z* (%) 255 (100) [M^+^], 240 (26); HRMS: 256.07894 (calcd. 256.07906) [M^+^ + H].

Compound 2-[1-Cyano-2-(2-chlorophenyl-4-methoxyphenyl)-(*E*)-ethenyl)-thiophene (**1f**): analogously to the synthesis of **1c**, compound **1f** was obtained from 2′-chloro-2-methoxy-[1,1′-biphenyl]-4-carboxaldehyde (130 mg, 0.53 mmol), 2-thiopheneacetonitrile (56 μL, 0.53 mmol) and a catalytic amount of piperidine (eight drops via a Pasteur pipette) in ethanol (p.a., 2 mL). Yield: 55 mg (0.16 mmol, 30%); yellow solid of mp = 106 °C; *R*_f_ = 0.4 (ethyl acetate/*n*-hexane, 1:9); *ν*_max_ (ATR)/cm^−1^: 3119, 2067, 3015, 2960, 2935, 2837, 2216, 1700, 1606, 1553, 1509, 1463, 1429, 1412, 1343, 1263, 1236, 1171, 1143, 1071, 1033, 1005, 894, 848, 829, 757, 734, 699, 638, 619; ^1^H NMR (300 MHz, CDCl_3_): δ 3.89 ppm (3 H, s, OCH_3_), 7.11 (1 H, dd, J = 5.2, 3.8 Hz, thienyl-4H), 7.3–7.4 (5 H, m, Ph/thienyl-H), 7.4–7.5 (4 H, m, Ph/thienyl-H), 7.64 (1 H, s, CH); ^13^C NMR (75 MHz, CDCl_3_): δ 55.8 (OCH_3_), 106.1 (*C*-CN), 110.5, 117.0 (CN), 122.3, 126.3, 127.3, 128.1, 128.9, 129.4, 129.9, 131.4, 133.7, 134.4, 136.8, 139.3, 157.0 (*C*-OCH_3_); *m*/*z* (%) 351 (100) [M^+^], 320 (11), 301 (16), 285 (11), 274 (11), 241 (41), 226 (9), 210 (11), 196 (12), 150 (25).

Compound 2-[1-Cyano-2-[4-Methoxy-3-(2-methoxyethoxy)phenyl]-(*E*)-ethenyl}-thiophene (**1g**): analogously to the synthesis of **1c**, compound **1g** was obtained from 4-methoxy-3-(2-methoxyethoxy)benzaldehyde (360 mg, 1.71 mmol), 2-thiopheneacetonitrile (182 μL, 1.71 mmol) and a catalytic amount of piperidine (four drops via a Pasteur pipette) in ethanol (p.a., 5 mL). Yield: 245 mg (0.78 mmol, 46%); yellow gum; *R*_f_ = 0.4 (ethyl acetate/*n*-hexane, 1:1); *ν*_max_ (ATR)/cm^−1^: 3096, 3085, 2994, 2942, 2879, 2844, 2819, 2212, 1594, 1576, 1524, 1510, 1486, 1464, 1436, 1342, 1330, 1273, 1255, 1201, 1150, 1122, 1017, 964, 893, 837, 805, 837, 805, 729, 632, 613, 584; ^1^H NMR (300 MHz, CDCl_3_): δ 3.47 (3 H, s, OCH_3_), 3.8–3.9 (2 H, m, CH_2_), 3.93 (3 H, s, OCH_3_), 4.2–4.3 (2 H, m, CH_2_), 6.92 (1 H, d, J = 8.5 Hz, Ph-5H), 7.07 (1 H, dd, J = 5.1, 3.7 Hz, thienyl-4H), 7.2–7.4 (4 H, m, Ph/thienyl-H), 7.64 (1 H, s, CH); ^13^C NMR (75 MHz, CDCl_3_): δ 56.0 (OCH_3_), 59.2 (OCH_3_), 68.5 (CH_2_), 70.8 (CH_2_), 103.4 (*C*-CN), 111.4, 112.8, 117.4 (CN), 124.5, 125.6, 126.5, 128.0, 139.6, 139.7, 148.5 (*C*-OC), 151.8 (*C*-OC); *m*/*z* (%) 315 (100) [M^+^], 257 (85), 242 (8), 224 (11), 214 (11), 196 (47), 185 (8), 140 (10), 59 (99), 45 (13).

Compound 2-{1-Cyano-2-[3,4-bis(2-methoxyethoxy)phenyl]-(*E*)-ethenyl}-thiophene (**1h**): analogously to the synthesis of **1c**, compound **1h** was obtained from 3,4-bis(2-methoxyethoxy)benzaldehyde (885 mg, 3.3 mmol), 2-thiopheneacetonitrile (350 μL, 3.3 mmol) and a catalytic amount of piperidine (eight drops via a Pasteur pipette) in ethanol (p.a., 10 mL). Yield: 383 mg (1.07 mmol, 32%); brown gum; *R*_f_ = 0.6 (ethyl acetate/*n*-hexane, 1:1); *v*_max_ (ATR)/cm^−1^: 3105, 2988, 2927, 2890, 2822, 2211, 1590, 1570, 1509, 1448, 1433, 1375, 1328, 1272, 1232, 1195, 1181, 1149, 1120, 1050, 1028, 929, 895, 860, 844, 793, 699, 636, 614, 588; ^1^H NMR (300 MHz, CDCl_3_): δ 3.47 (3 H, s, OCH_3_), 3.48 (3 H, s, OCH_3_), 3.8–3.9 (4 H, m, 2 × CH_2_), 4.2–4.3 (4 H, m, 2 × CH_2_), 6.96 (1 H, d, J = 8.5 Hz, Ph-5H), 7.07 (1 H, dd, J = 5.2, 3.6 Hz, thienyl-4H), 7.2–7.4 (3 H, m, Ph/thienyl-H), 7.61 (1 H, s, CH); ^13^C NMR (75 MHz, CDCl_3_): δ 59.2 (OCH_3_), 59.3 (OCH_3_), 68.7 (CH_2_), 68.9 (CH_2_), 70.9 (CH_2_), 103.5 (*C*-CN), 113.8, 113.9, 117.3 (CN), 124.3, 125.6, 126.5, 126.8, 128.0, 139.6, 148.9 (*C*-OC), 151.2 (*C*-OC); *m*/*z* (%) 359 (100) [M^+^], 301 (33), 283 (23), 269 (36), 243 (34), 196 (22), 59 (100), 43 (15).

Compound 2-[1-Cyano-2-(4-*N*-methylpiperazin-1-ylphenyl)-(*E*)-ethenyl]-thiophene (**1i**): analogously to the synthesis of **1c**, compound **1i** was obtained from 4-(4-methylpiperazin-1-yl)benzaldehyde (674 mg, 3.3 mmol), 2-thiopheneacetonitrile (350 μL, 3.3 mmol) and a catalytic amount of piperidine (eight drops via a Pasteur pipette) in ethanol (p.a., 10 mL). The formed precipitate was collected, washed with ethanol, and dried in a vacuum. Yield: 430 mg (1.39 mmol, 42%); yellow-orange solid of mp 161 °C; *ν*_max_ (ATR)/cm^−1^: 3067, 2938, 2846, 2798, 2753, 2204, 1604, 1578, 1520, 1508, 1446, 1430, 1379, 1355, 1293, 1237, 1191, 1164, 1143, 1081, 1055, 1006, 940, 921, 891, 848, 816, 710, 692, 644, 627, 605, 566; ^1^H NMR (300 MHz, CDCl_3_): δ 2.38 (3 H, s, CH_3_), 2.5–2.6 (4 H, m, 2 × CH_2_), 3.3–3.4 (4 H, m, 2 × CH_2_), 6.9–7.0 (2 H, m, Ph-3H/5H), 7.07 (1 H, dd, J = 5.2, 3.7 Hz, thienyl-4H), 7.2–7.3 (3 H, m, thienyl-H, CH), 7.8–7.9 (2 H, m, Ph-2H/6H); ^13^C NMR (75 MHz, CDCl_3_): δ 46.2 (CH_3_), 47.5 (CH_2_), 54.8 (CH_2_), 107.4 (*C*-CN), 114.5, 117.8 (CN), 124.9, 125.7, 127.9, 130.9, 139.8, 152.3 (*C*-NC); *m*/*z* (%) 309 (100) [M^+^], 265 (11), 238 (48), 224 (38), 209 (32), 177 (13), 166 (10), 71 (76), 56 (10), 43 (97).

Compound 2-[1-Cyano-2-(3-fluoro-4-methoxyphenyl)-(*E*)-ethenyl]-thiophene (**1j**): analogously to the synthesis of **1c**, compound **1j** was obtained from 3-fluoro-4-methoxybenzaldehyde (509 mg, 3.3 mmol), 2-thiopheneacetonitrile (350 μL, 3.3 mmol) and a catalytic amount of piperidine (eight drops via a Pasteur pipette) in ethanol (p.a., 10 mL). Yield: 260 mg (1.00 mmol, 30%); yellow solid of mp 95 °C; *R*_f_ = 0.7 (ethyl acetate/*n*-hexane, 1:3); *ν*_max_ (ATR)/cm^−1^: 3104, 3030, 2983, 2956, 2923, 2848, 2591, 2553, 2274, 2214, 2034, 1890, 1614, 1572, 1510, 1467, 1443, 1420, 1388, 1349, 1311, 1284, 1246, 1155, 1131, 1021, 982, 908, 851, 808, 766, 749, 676, 633, 608, 589; ^1^H NMR (300 MHz, CDCl_3_): δ 3.96 (3 H, s, OCH_3_), 7.0–7.1 (2 H, m, Ph-5H, thienyl-4H), 7.2–7.4 (3 H, m, Ph/thienyl-H, CH), 7.6–7.7 (2 H, m, Ph/thienyl-H); ^13^C NMR (75 MHz, CDCl_3_): δ 56.3 (OCH_3_), 104.8 (*C*-CN), 113.3, 116.5 (CN), 126.0, 127.0, 128.1, 137.9, 138.0, 139.1, 149.3 (*C*-OCH_3_), 150.5–153.7 (m, CF); *m*/*z* (%) 260 (53) [M^+^ + 1], 259 (100) [M^+^], 244 (68), 228 (25), 214 (26), 196 (89), 189 (34), 172 (26), 145 (15), 45 (27).

Compound 2-{1-Cyano-2-[3-fluoro-4-(2-methoxyethoxy)phenyl]-(*E*)-ethenyl}-thiophene (**1k**): analogously to the synthesis of **1c**, compound **1k** was obtained from 3-fluoro-4-(2-methoxyethoxy)benzaldehyde (85 mg, 0.43 mmol), 2-thiopheneacetonitrile (46 μL, 0.43 mmol) and a catalytic amount of piperidine (one drop via a Pasteur pipette) in ethanol (p.a., 2 mL). Yield: 48 mg (0.16 mmol, 37%); yellow solid of mp 65 °C; *R*_f_ = 0.8 (ethyl acetate/*n*-hexane, 1:3); *ν*_max_ (ATR)/cm^−1^: 3118, 3076, 2937, 2894, 2854, 2826, 2214, 2078, 1616, 1574, 1523, 1509, 1442, 1430, 2345, 1315, 1280, 1198, 1135, 1120, 1078, 1056, 1067, 983, 963, 891, 866, 853, 829, 796, 719, 634, 607, 595; ^1^H NMR (300 MHz, CDCl_3_): δ 3.47 (3 H, s, OCH_3_), 3.7–3.9 (2 H, m, CH_2_), 4.2–4.3 (2 H, m, CH_2_), 7.03 (1 H, d, J = 8.5 Hz, Ph-5H), 7.0–7.1 (1 H, m, thienyl-4H), 7.2–7.4 (3 H, m, Ph/thienyl-H, CH), 7.5–7.7 (2 H, m, Ph/thienyl-H); ^13^C NMR (75 MHz, CDCl_3_): δ 59.3 (OCH_3_), 69.0 (CH_2_), 70.8 (CH_2_), 104.9 (*C*-CN), 114.9, 116.5 (CN), 126.1, 126.2, 127.0, 128.1, 138.0, 139.1, 148.8 (*C*-OCH_3_), 150.7–154.0 (m, CF); *m*/*z* (%) 303 (99) [M^+^], 245 (100), 228 (13), 196 (15), 59 (99), 45 (12).

Compound 2-[1-Cyano-2-(3-bromo-4-methoxyphenyl)-(*E*)-ethenyl]-thiophene (**1l**): analogously to the synthesis of **1c**, compound **1l** was obtained from 3-bromo-4-methoxybenzaldehyde (710 mg, 3.3 mmol), 2-thiopheneacetonitrile (350 μL, 3.3 mmol) and a catalytic amount of piperidine (eight drops via a Pasteur pipette) in ethanol (p.a., 10 mL). The crude product was purified by column chromatography (silica gel 60) followed by recrystallisation from EtOH/*n*-hexane. Yield: 325 mg (1.02 mmol, 31%); yellow solid of mp 124–125 °C; *R*_f_ = 0.54 (ethyl acetate/*n*-hexane, 1:3); *ν*_max_ (ATR)/cm^−1^: 3109, 3014, 2972, 2944, 2838, 2213, 1598, 1581, 1557, 1518, 1495, 1462, 1439, 1431, 1401, 1364, 1344, 1305, 1288, 1264, 1246, 1223, 1200, 1184, 1165, 1092, 1051, 1012, 946, 902, 864, 845, 828, 804, 775, 764, 743, 699, 670; ^1^H NMR (300 MHz, CDCl_3_): δ 3.94 (3 H, s, OCH_3_), 6.95 (1 H, d, J = 8.7 Hz, Ph-5H), 7.0–7.1 (1 H, m, thienyl-4H), 7.2–7.4 (3 H, m, Ph/thienyl-H), 7.9–8.0 (2 H, m, Ph-2H, CH); ^13^C NMR (75 MHz, CDCl_3_): δ 56.4 (OCH_3_), 104.9 (*C*-CN), 111.9, 112.2, 116.8 (CN), 126.1, 127.0, 127.4, 128.1, 129.2, 134.6, 137.5, 139.1, 157.5 (*C*-OCH_3_); *m*/*z* (%) 321 (100) [M^+^], 319 (99) [M^+^], 306 (16), 304 (15), 196 (82).

Compound 2-[1-Cyano-2-(3-hydroxyphenyl)-(*E*)-ethenyl]-thiophene (**1m**)**:** analogously to the synthesis of **1c**, compound **1m** was obtained from 3-hydroxybenzaldehyde (403 mg, 3.3 mmol), 2-thiopheneacetonitrile (350 μL, 3.3 mmol) and a catalytic amount of piperidine (eight drops via a Pasteur pipette) in ethanol (p.a., 10 mL). Yield: 230 mg (1.01 mmol, 31%); yellow solid of mp 94–96 °C; *R*_f_ = 0.42 (ethyl acetate/*n*-hexane, 1:3); *ν*_max_ (ATR)/cm^−1^: 3371, 3071, 3024, 2225, 1602, 1578, 1498, 1451, 1426, 1378, 1358, 1327, 1276, 1236, 1173, 1164, 1081, 1053, 1001, 987, 965, 903, 875, 850, 828, 782, 750, 721, 696, 679, 634; ^1^H NMR (300 MHz, CDCl_3_): δ 4.4–4.8 (1 H, br s, OH), 6.9–7.0 (1 H, m, Ph-2H), 7.0–7.1 (1 H, m, thienyl-4H), 7.2–7.4 (5 H, m, Ph/thienyl-H), 7.44 (1 H, s, CH); ^13^C NMR (75 MHz, CDCl_3_): δ 106.1 (*C*-CN), 114.7, 116.9, 118.0 (CN), 122.4, 126.4, 127.3, 128.1, 130.3, 134.6, 138.9, 139.5, 156.1 (COH); *m*/*z* (%) 227 (100) [M^+^], 210 (34), 194 (38), 183 (36).

Compound 2-{1-Cyano-2-[4-methoxy-3-(3-morpholinopropoxy)phenyl]-(*E*)-ethenyl}-thiophene (**1n**): analogously to the synthesis of **1c**, compound **1n** was obtained from 4-methoxy-3-(3-morpholinopropoxy)benzaldehyde (307 mg, 1.1 mmol), 2-thiopheneacetonitrile (117 μL, 1.1 mmol) and a catalytic amount of piperidine (five drops via a Pasteur pipette) in ethanol (p.a., 10 mL). Yield: 130 mg (0.35 mmol, 32%); yellow brown gum; *R*_f_ = 0.5 (5 % MeOH in CH_2_Cl_2_); *ν*_max_ (ATR)/cm^−1^: 3106, 2955, 2855, 2812, 2246, 2212, 1729, 2684, 2589, 1509, 1433, 1329, 1265, 1142, 1115, 1022, 907, 862, 848, 805, 727, 698, 633, 613, 587; ^1^H NMR (300 MHz, CDCl_3_): δ 2.0–2.1 (2 H, m, CH_2_), 2.4–2.5 (4 H, m, 2 × CH_2_), 2.5–2.6 (2 H, m, CH_2_), 3.7–3.8 (4 H, m, 2 × CH_2_), 3.93 (3 H, s, OCH_3_), 4.19 (2 H, t, J = 6.6 Hz, CH_2_), 6.92 (1 H, d, J = 8.5 Hz, Ph-5H), 7.07 (1 H, dd, J = 5.1, 3.7 Hz, thienyl-4H), 7.2–7.4 (4 H, m, Ph/thienyl-H), 7.61 (1 H, s, CH); ^13^C NMR (75 MHz, CDCl_3_): δ 26.2 (CH_2_), 53.7 (CH_2_), 55.4 (CH_2_), 56.0 (OCH_3_), 67.0 (CH_2_), 67.3 (CH_2_), 103.3 (*C*-CN), 111.4, 112.3, 117.4 (CN), 124.1, 125.5, 126.4, 128.0, 139.7, 148.5 (*C*-OC), 151.6 (*C*-OC); *m*/*z* (%) 385 (27) [M^+^ + 1], 384 (100) [M^+^], 269 (29), 196 (38), 128 (66), 100 (98), 87 (25), 70 (78), 56 (57), 42 (66).

Compound 2-{1-Cyano-2-[*N*-methylindol-5-yl]-(*E*)-ethenyl}-thiophene (**1o**): analogously to the synthesis of **1c**, compound **1o** was obtained from *N*-methylindole-5-carboxaldehyde (413 mg, 2.6 mmol), 2-thiopheneacetonitrile (276 μL, 2.6 mmol) and a catalytic amount of piperidine (eight drops via a Pasteur pipette) in ethanol (p.a., 10 mL). After cooling to room temperature, the reaction mixture was kept at 4 °C for 24 h and the formed crystals were collected, washed with ethanol, and dried in vacuum. Yield: 210 mg (0.79 mmol, 31%); yellow brown crystals of mp 133–134 °C; *R*_f_ = 0.38 (ethyl acetate/*n*-hexane, 1:3); *ν*_max_ (ATR)/cm^−1^: 3108, 3014, 2937, 2213, 1611, 1591, 1568, 1508, 1484, 1433, 1420, 1388, 1368, 1333, 1310, 1246, 1225, 1166, 1153, 1101, 1080, 1056, 907, 844, 827, 761, 729, 686, 633, 620; ^1^H NMR (300 MHz, CDCl_3_): δ 3.80 (3 H, s, NCH_3_), 6.5–6.6 (1 H, m, indole-3H), 7.0–7.1 (2 H, m, indole-2H, thienyl-4H), 7.2–7.4 (3 H, m, indole/thienyl-H), 7.50 (1 H, s, CH), 7.82 (1 H, d, J = 8.7 Hz, indole-7H), 8.12 (1 H, s, indole-4H); ^13^C NMR (75 MHz, CDCl_3_): δ 33.0 (NCH_3_), 102.3 (indole-3C), 109.8 (*C*-CN), 117.8 (CN), 122.7, 123.4, 125.0, 125.1, 125.9, 127.9, 128.7, 130.3, 137.8, 140.2, 141.9; *m*/*z* (%) 264 (46) [M^+^], 248 (62), 231 (84), 216 (46), 125 (35).

### 4.4. Biological Evaluations

#### 4.4.1. Cell Lines and Culture Conditions

The well-differentiated p53 wild-type human hepatoblastoma cell line HepG2 (ATCC# 8065) and the highly differentiated p53 mutated human hepatocellular carcinoma cell line Huh-7 (JCRB#0403) were grown in RPMI 1640 medium supplemented with 10% fetal bovine serum and 100 U/mL penicillin and 100 mg/mL streptomycin. Murine AML-12 hepatocytes were obtained from ATCC (CRL-2254) and grown in DMEM/F-12 Glutamax medium supplemented with 10% fetal bovine serum, 10 µg/mL insulin, 5.5 µg/mL transferrin, 5 ng/mL selenium (Gibco ITS-G 100X) and 40 ng/mL dexamethasone (Sigma-Aldrich). All cell lines were incubated in a 37 °C, 5% CO_2_, 95% humidified atmosphere. 

#### 4.4.2. Determination of Growth Inhibition

Treatment-induced changes in cell number were evaluated by crystal violet staining, as previously described [50]. Briefly, 1.500 cells per well were seeded in 96-well plates for 24 h at 37 °C, 5% CO_2_. For the experiments with HepG2 and Huh-7 cell lines, cells were incubated with rising concentrations (0–20 µM) of the new compounds (**1a**–**1o**) for 48 h. AML12-cells were treated with compounds **1a**, **1b**, **1c**, **1e**, **1i** and **1j** for 48 h and at concentrations ranging from 0.5–64 µM. Thereafter, the cells were fixed with 1% glutaraldehyde and stained with 0.1% crystal violet (Sigma-Aldrich). The unbound dye was removed by rinsing with water. Bound crystal violet was solubilized with 0.2% Triton X-100 (Sigma-Aldrich). Light extinction of crystal violet, which increases linearly with cell number, was analyzed at 570 nm using an ELISA-Reader (Dynex Technologies, Denkendorf, Germany). All experiments were carried out in duplicate or triplicate [51].

#### 4.4.3. Measurement of Apoptosis-Specific Caspase-3 Activity

Changes in caspase-3 activity were measured by the cleavage of the fluorogenic substrate AC-DEVD-AMC (EMD Millipore, Billerica, MA, USA), as described previously [52]. After 24 h of incubation with **1a**, **1b**, **1c**, **1e** and sorafenib (1 and 10 µM) the cells were harvested and lysed with lysis buffer. Subsequently, the lysates were incubated for 1 h at 37 °C with a substrate solution containing 20 μg/mL AC-DEVD-AMC, 20 mM HEPES, 10% glycerol and 2 mM DTT at pH 7.5. Substrate cleavage was measured fluorometrically using a Varioskan Flash Fluorometer (Thermo Fisher Scientific, Waltham, MA, USA; filter sets: ex 360/40 nm, em 460/10 nm). *n* = 3 independent measurements were performed in triplicate, and data are given as the mean percentage increase ± SD above control, which was set 100%.

#### 4.4.4. Colony-Formation Assay

HepG2 cells were seeded in 6-well plates at a density of 300 cells/well and allowed to attach overnight. After treatment with test compounds (0 µM/control, 1 µM, 5 µM, and 10 µM), colony formation and growth were observed for 2 weeks. Then, the plates were washed twice with PBS, fixed with 4% formaldehyde for 1 h, and stained with 0.5% crystal violet for 3 min. Excessive crystal violet was washed out and the colonies were counted under the microscope. A colony was defined as a cell aggregate with 50 or more cells [30]. Representative images were taken with a kappa digital camera system (Kappa Optronics GmbH, Germany). A total of *n* = 3 experiments were performed, and significance was tested by one-way ANOVA assays.

#### 4.4.5. LDH Assay

Unspecific cytotoxicity induced by treatment with 0.5 µM and 10 µM of **1a**, **1b**, **1c** and sorafenib (LC Laboratories, Woburn, MA, USA) was determined by measuring the release of lactate dehydrogenase (LDH) from HepG2 cells into the supernatant after 12 and 24 h (Cytotoxicity Detection KitPLUS LDH, Roche Diagnostics GmbH, Mannheim, Germany) [52]. The supernatant of treated samples was collected, and adherent non-treated cells were lysed with 2% Triton X-100 for 10 min to determine the maximum LDH release. All samples were mixed with catalyst and dye solution for 30 min, resulting in the formation of formazan dye proportional to LDH enzyme activity. The absorbance was measured at 490/630 nm using an ELISA reader (Dynex Technologies, Denkendorf, Germany) and cytotoxicity was determined by subtracting the percentage of LDH release under control conditions of those from treated samples. Experiments were performed in duplicate and data are given as mean percentage changes ± SD as compared to controls of *n* = 3 experiments.

#### 4.4.6. Enzymatic Kinase Assay

The kinase inhibiting potency of compound **1a**, **1b** and **1c** (10 µM) was screened in a cell-free kinase assay, which consisted of a custom panel of 43 human protein kinases for **1c** (listed in Figure 5A), as well as a subpanel of six kinases for **1a** and **1b** (Figure 5B). The initial panel was chosen to screen the lead compound **1c** based on the union of two strategies. A total of 29 kinases were selected based on the correlated results of three in silico target prediction methods including structure- as well as ligand-based techniques and 20 kinases because of their relevance for HCC (note that six kinases were part of both strategies) [53,54,55]. In a second round, compounds **1a** and **1b** were also screened against the subpanel of six kinases that were most strongly inhibited by compound **1c**, namely VEGFR-2, VEGFR-1, Pim-1, CLK1, c-KIT and Pim-2.

The assay was performed by Eurofins KinaseProfilerTM service (Eurofins, France) and the determination of enzymatic activity was assessed as previously described [56]. Additionally, an IC_50_ determination of compound **1c** against the VEGFR-2 was performed with a concentration range of 0.003 to 30 µM.

#### 4.4.7. In Vivo Evaluation of Antineoplastic Effects Using the Chorioallantoic Membrane (CAM) Assay

The chorioallantoic membrane (CAM) assay was performed as described previously [57]. Fertilized chicken eggs (*Gallus gallus*) were obtained from commercial sources (Valo GmbH, Germany) and incubated in a humid environment at 38 °C. On day 3 of the egg development, a window was cut in the eggshell to access the developing vascular network of the CAM. At day 10 of chicken embryo development, a sterile silicon ring (10 mm diameter) was placed on the CAM and tumor plugs of 3 × 10^6^ HepG2 cells in 20 µL Matrigel (BD Biosciences) with reduced growth factors were placed inside the silicon ring. After 24 h at 38 °C in an incubator, the tumor plaques attached and connected to the (tumor-)feeding CAM microvasculature. Then, the tumor plaques were topically treated with either PBS (negative control) or PBS containing rising concentrations of **1b** (0–10 µM), **1c** (0–2.5 µM) or sorafenib (10 μM) for 3 days. Tumor growth and viability of the embryo were controlled daily by stereo microscopy. After 72 h of treatment, the tumors were recovered for weighing and pictures were taken using a stereomicroscope equipped with a Kappa digital camera system (Kappa Optronics GmbH, Germany). For data analysis, the tumor weight of **1c**-treated and untreated control tumors was determined and the mean weight of treated vs. untreated tumors of 11 eggs for each condition was calculated. Statistical significance evaluated by one-way ANOVA tests.

### 4.5. Computational Evaluation

#### 4.5.1. Structure Selection

The kinase centered KLIFS database [58] (accessed November 2020) was searched for available X-ray structures of human VEGFR-2. Restricting the search to human structures with a ligand bound having a KLIFS quality score above 6 and resolution below 2 Å resulted in 13 structures, all of which happened to be in DFG-out conformation. After further inspections, structure 3VHE [33] was chosen because it contains no mutation and has the best resolution (1.55 Å).

#### 4.5.2. Molecular Docking

To generate docking poses for the compounds of interest, we used SeeSAR version 10.2 (www.biosolveit.de/SeeSAR). The VEGFR-2 structure 3VHE was chosen as the target and the binding pocket defined by the co-crystallized ligand. The docking library contained all compounds tested in the enzymatic assay for VEGFR-2 inhibition (**1a**, **1b**, **1c**). Default parameters were used for docking and up to 500 poses per compound were generated. In SeeSAR, all poses were evaluated with the built-in HYDE scoring function [59]. The top scoring poses and estimated affinities were further analyzed. For lead compound **1c**, the top scoring poses exhibited a stable orientation of the scaffold with mainly three variations in the orientations of the rings, all within small score distances. Pose 11 was finally chosen, because it was best suited for the subsequent MD simulations. For the other two compounds, high scoring poses with orientations similar to the chosen pose for **1c** were favored. For compounds **1a**, this happened to be the highest scoring pose, whereas for **1b** it was the sixth highest scoring pose. Selected docking poses were energy minimized using the MMFF94s force field [34] and visually analyzed in LigandScout 4.4 (license kindly provided by Prof. G. Wolber) [35].

#### 4.5.3. Molecular Dynamics Simulation

The VEGFR-2 structure 3VHE was retrieved from the protein data bank [60]. The structure was prepared with OESpruce (OpenEye toolkit 2020.1.0) by modeling missing atoms and residues, adding caps, as well as protonation at pH 7.4.

The prepared protein and the selected docking pose of compound **1c** were prepared for MD simulation in Maestro 12.3 (Schrödinger Suite 2020-1) by solvating the complex in a cubic SPC water box with 10 Å padding and 0.15 M KCl. The prepared system was subjected to 100 ns of unrestrained MD simulation using Desmond 6.1 (Schrödinger Suite 2020-1). Coordinates were saved every 100 ps resulting in 1000 frames for analysis. The resulting trajectory was aligned on the protein backbone of the first frame and subsequently analyzed using VMD 1.9.3 [61].

## Figures and Tables

**Table 1:** Anti-proliferative activity expressed as IC_50_ (µM)^1^ of test compounds **1a**–**o** and **2** against human HepG2 or Huh-7 hepatoma cell lines and non-malignant murine AML-12 hepatocytes after 48 h (sorafenib was used as positive control).

Compounds	HepG2	Huh-7	AML-12
**1a**	1.46 ± 0.21	1.2 ± 0.42	6.79 ± 0.32
**1b**	0.72 ± 0.09	0.67 ± 0.13	2.72 ± 0.78
**1c**	0.55 ± 0.26	0.32 ± 0.15	1.81 ± 0.31
**1d**	>20	>20	-
**1e**	1.30 ± 0.17	1.85 ± 0.21	14.78 ± 2.39
**1f**	>20	>20	-
**1g**	>20	>20	-
**1h**	>20	>20	-
**1i**	9.30 ± 0.82	11.79 ± 0.56	20.53 ± 1.25
**1j**	4.90 ± 1.54	5.70 ± 1.20	9.42 ± 0.91
**1k**	>20	>20	-
**1l**	13.87 ± 1.16	-	-
**1m**	>20	>20	-
**1n**	>20	>20	-
**1o**	>20	>20	-
**2**	6.26 ± 1.18	-	-
**Sorafenib**	2.73 ± 0.76	2.50 ± 0.14	-

^1^ Concentration where 50% inhibition of proliferation was observed, as determined after 48 h of incubation (mean ± SD), *n* = 2 experiments for insensitive derivatives (IC_50_ value >20 µM) and *n* = 3 for sensitive compounds.

## Data Availability

The data presented in this study are available on request from the corresponding author.

## Abbreviations

CAM	Chorioallantoic membrane
CLK1	Cdc2-like kinase-1
EGFR	Epidermal growth factor receptor
FGFR	Fibroblast growth factor receptor
GIST	Gastrointestinal stromal tumor
HCC	Hepatocellular carcinoma
IGF-1R	Insulin-like growth factor 1-receptor
LDH	Lactate dehydrogenase
PDGFR	Platelet-derived growth factor receptor
Pim	Proviral integration site for Moloney murine leukemia virus
RCC	Renal cell carcinoma
VEGFR	Vascular endothelial growth factor receptor
